# Draft Genome Sequence of *Pseudomonas stutzeri* Strain 19, an Isolate Capable of Efficient Degradation of Aromatic Hydrocarbons

**DOI:** 10.1128/genomeA.01373-17

**Published:** 2017-12-07

**Authors:** Lisa M. Brown, Thusitha S. Gunasekera, Oscar N. Ruiz

**Affiliations:** a Environmental Microbiology Group, University of Dayton Research Institute, University of Dayton, Dayton, Ohio, USA; b Fuels and Energy Branch, Aerospace Systems Directorate, Air Force Research Laboratory, Wright-Patterson AFB, Ohio, USA

## Abstract

*Pseudomonas stutzeri* strain 19 is a Gram-negative bacterium capable of degrading aromatic hydrocarbons. The draft genome of *P. stutzeri* 19 is estimated to be 5.1 Mb, containing 4,652 protein-coding genes and a G+C content of 63.3%. Multiple genes responsible for the degradation of aromatics are present in this strain.

## GENOME ANNOUNCEMENT

*Pseudomonas stutzeri* strain 19 was isolated from a wastewater sample from Dayton, OH, USA. *P. stutzeri* 19 was shown, through gas chromatography-mass spectrometry (GC-MS) analysis, to efficiently metabolize toluene, xylenes, and 1,2,4-trimethyl benzene. Comparative BLAST analysis (http://blast.ncbi.nlm.nih.gov/Blast.cgi) of the 16S rRNA gene of *P. stutzeri* 19, identified using RNAmmer (1), showed 99% similarity with *P. stutzeri* DSM 4166 and *P. stutzeri* A1501, while Rapid Annotations using Subsystems Technology (RAST) identified *P. stutzeri* A1501 as the closest neighbor, with a score of 507. The genome of *P. stutzeri* was chosen for sequencing due to its ability to degrade recalcitrant aromatics and grow in harsh hydrocarbon-containing environments.

Whole-genome shotgun sequencing was performed on a Roche 454-GS Junior platform, producing 334,879 reads. Newbler assembly (version 2.9) was used to align reads, creating 136 large (>500-bp) contigs with an average size of 37,500 bp, an *N*_50_ of 104,286, and an *L*_50_ of 15. The draft genome sequence was 5,100,040 bp in length, with a G+C content of 63.3%. The largest contig extended for 263,585 bp. The NCBI Prokaryotic Genome Annotation Pipeline (PGAP) (http://www.ncbi.nlm.nih.gov/genome/annotation_prok/) predicted 4,895 genes, 4,652 coding sequences (CDSs), and 54 tRNAs. Rapid genome annotations using the RAST server (2) assigned the protein-coding sequences to 512 subsystems, of which amino acids and derivatives (*n* = 439 CDSs), carbohydrates (*n* = 368), cofactors, vitamins, prosthetic groups, and pigments (*n* = 332), protein metabolism (*n* = 278), fatty acids, lipids, and isoprenoids (*n* = 161), RNA metabolism (*n* = 204), nucleosides and nucleotides (*n* = 115), virulence, disease, and defense (*n* = 129), stress response (*n* = 176), respiration (*n* = 148), DNA metabolism (*n* = 162), motility and chemotaxis (*n* = 129), membrane transport (*n* = 200), and cell wall and capsule (*n* = 183) were most abundant.

The NCBI PGAP predicted multiple genes involved in hydrocarbon degradation, including catechol 1,2-dioxygenase, homogentisate 1,2-dioxygenase, phenol monooxygenase, small and large subunits of benzoate 1,2-dioxygenase (*benA* and* benB*), alkane 1-monooxygenease, rubredoxin, alkene reductase, 2-alkenal reductase, P450, and a benzoate transporter protein, among others. BLAST analysis revealed two coding sequences with 99% homology to the alpha and beta subunits of toluene 1,2-dioxygenase of *P. putida* MT53 plasmid pWW53. Also, coding sequences with 96% homology to the xylene monooxygenase electron transfer subunit and 98% homology to the xylene monooxygenase hydrolase subunit of *P. putida* MT53 plasmid pWW53 were found. The presence of these enzymes explains the toluene and xylene degradation capacities of *P. stutzeri* 19. The genes for protocatechuate 3,4-dioxygenase, 3-carboxymuconate cycloisomerase, and 4-carboxymuconolactone decarboxylase of the central protocatechuate catabolic pathway for aromatic degradation were also present. A cluster of genes was observed with at least 78% homology to the *ttg*2 operon of *P. putida* that encodes an ABC transporter implicated in resistance to toluene (3, 4). The genome of *P. stutzeri* 19 encodes many multidrug and heavy-metal resistance-nodulation-division (RND) efflux transporters, some of which have been associated with hydrocarbon resistance (5). The genome of *P. stutzeri* strain 19 will help to understand the adaptive mechanisms deployed by Gram-negative bacteria for survival and proliferation in hydrocarbons.

### Accession number(s).

This whole-genome shotgun project has been deposited at DDBJ/ENA/GenBank under the accession number NFZU00000000. The version described in this paper is NFZU01000000.
